# Numerical Algorithms for Estimating Probability Density Function Based on the Maximum Entropy Principle and Fup Basis Functions

**DOI:** 10.3390/e23121559

**Published:** 2021-11-23

**Authors:** Nives Brajčić Kurbaša, Blaž Gotovac, Vedrana Kozulić, Hrvoje Gotovac

**Affiliations:** Faculty of Civil Engineering, University of Split, Architecture and Geodesy, 21000 Split, Croatia; blaz.gotovac@gradst.hr (B.G.); vedrana.kozulic@gradst.hr (V.K.); hrvoje.gotovac@gradst.hr (H.G.)

**Keywords:** maximum entropy, Shannon entropy, Fup basis functions, probability density function

## Abstract

Estimation of the probability density function from the statistical power moments presents a challenging nonlinear numerical problem posed by unbalanced nonlinearities, numerical instability and a lack of convergence, especially for larger numbers of moments. Despite many numerical improvements over the past two decades, the classical moment problem of maximum entropy (MaxEnt) is still a very demanding numerical and statistical task. Among others, it was presented how Fup basis functions with compact support can significantly improve the convergence properties of the mentioned nonlinear algorithm, but still, there is a lot of obstacles to an efficient pdf solution in different applied examples. Therefore, besides the mentioned classical nonlinear Algorithm 1, in this paper, we present a linear approximation of the MaxEnt moment problem as Algorithm 2 using exponential Fup basis functions. Algorithm 2 solves the linear problem, satisfying only the proposed moments, using an optimal exponential tension parameter that maximizes Shannon entropy. Algorithm 2 is very efficient for larger numbers of moments and especially for skewed pdfs. Since both Algorithms have pros and cons, a hybrid strategy is proposed to combine their best approximation properties.

## 1. Introduction

Many physical processes cannot be characterized deterministically owing to the presence of intrinsic or parametric uncertainty due to their physical nature, interpretation or measurements. Therefore, results are usually given in the form of a certain number of the first few statistical power moments or, rarely, as a probability density function (pdf) [1,2,3].

The maximum entropy principle, defined by Jaynes [4], is a versatile tool for statistical inference of the probability density function from its moments by maximizing Shannon entropy [5]. The principle states that among all possible pdfs that satisfy our incomplete information about the system, the one that maximizes entropy is the least biased estimate that can be made. It agrees with everything that is known but carefully avoids anything that is unknown [6,7].

In the past few decades, a great number of maximum entropy algorithms (MEAs) have been developed and applied in various fields of science, such as continuum mechanics [8], signal processing [9,10], chemical engineering [11,12,13] and heat transfer [14]. In coastal engineering, a MEAs are used as a powerful tool for the prediction of extreme significant wave heights [15,16,17] and for the distribution of streamwise velocity in open channels [18]. MEAs are especially popular in structural reliability analysis [19,20,21,22,23,24,25]. Their application goes much further, though; for example, MEAs used for the evaluation of the reliability of semiconductor devices [26]. MEAs have even been extended to multidimensional problems [27,28,29,30] and models in 2D domains and on surfaces [31].

MaxEnt algorithms for a higher number of moments are subjected to highly unbalanced nonlinearities, ill-conditioned Jacobian and Hessian matrices in Newton algorithms and many other numerical problems, such as insufficient arithmetic precision [3]. To overcome these difficulties, the MaxEnt algorithm uses orthogonal polynomials [2] and splines [32,33] instead of classic monomials.

Gotovac and Gotovac (2009) [34] employed a different and original idea using finite and localized basis functions with compact support closely related to algebraic polynomials, which made an efficient MaxEnt algorithm possible with more balanced nonlinearities and the ability to solve a higher number of moments. They used Fup basis functions of the second order with compact support, which are similar to wavelets and splines and belong to the class of atomic basis functions (ABF).

The history of the ABFs originates from V. L. and V. A. Rvachevs, [35]. The first monographs on the results of research were published in [36,37], while in [38,39] a detailed analysis of the current publications on ABF is provided, from the first publications until now. Furthermore, based on ABF theory, many different classes of weight functions have arisen, which are often used, especially in digital signal processing [40,41,42,43,44,45,46,47] and groundwater flow modeling [48,49,50,51]. A very interesting theory of atomic solitons is based on ABF theory that is used in new areas, like matter quantization, quantum gravity, Higgs fields, unified theory of nature, as well as in non-traditional areas, like medicine and life sciences, geology and financial markets [52,53,54,55].

In this paper, we will use two types of Fup basis functions that belong to the class of atomic basis functions (ABFs) [56]: the algebraic-type, ${\mathrm{Fup}}_{n}\left(x\right)$, and the exponential type, ${\mathrm{EFup}}_{n}\left(\omega ,x\right)$, containing an additional tension parameter, ω. While atomic basis functions of the algebraic type have been used for many years, ABFs of the exponential type are still not widely used [57]. This work uses Fup and EFup basis functions of the fourth order by, which monomials up to the fourth degree can be represented accurately. This approximation property of the basis functions affects the numerical precision of the requested pdf since it is the first few moments that give the greatest impact on the solution function of the MaxEnt problem.

Based on the algebraic ${\mathrm{Fup}}_{4}\left(x\right)$ and exponential ${\mathrm{EFup}}_{4}\left(x,\omega \right)$ basis functions, two Algorithms for solving the maximum entropy problem from known statistical moments are derived and presented. Algorithm 1 is a classical nonlinear algorithm [34], while Algorithm 2 is a fast linear algorithm. Exponential basis functions, ${\mathrm{EFup}}_{4}\left(x,\omega \right)$, use an unknown parameter, ω, that is selected considering the maximization of the Shannon entropy functional. Both Algorithms have their advantages and disadvantages, which will be demonstrated in various examples of the probability density functions.

## 2. Maximum Entropy Principle

Let f(x) be an unknown probability density function (pdf) defined in a finite real interval that satisfies a basic normalized zero statistical moment, which says that all pdf outcomes present a certain event:

(1) $$\overset{b}{\underset{a}{\int }}f\left(x\right)\mathrm{dx}=1,\;f\left(x\right)\geq 0\;\forall \;x\in \left[a,b\right]$$

Suppose that the additional moment constraints on f are given in the form of classical statistical moments of the higher order:

(2) $$\overset{b}{\underset{a}{\int }}x^{i}f\left(x\right)\mathrm{dx}=\mu_{i},\;\;\;i=1,\ldots ,m$$

where $\mu_{i}$ represents given and known statistical moments. Without losing generality, the interval [a,b] can be transformed into the interval [0,1], as we will write later in this article.

The estimation of the function, f(x), by the maximum entropy principle is obtained by maximizing the Shannon entropy, H(f), associated with the function, f, with constraints given by Equation (2), where the Shannon entropy is given in the form:

(3) $$H\left(f\right)=-\overset{1}{\underset{0}{\int }}f\left(x\right)\;\ln\left[f\left(x\right)\right]\mathrm{dx}$$

Jaynes [4] showed that maximizing H(f) with respect to f, under constraints (2), leads to the following analytical solution:

(4) $$f\left(x\right)=e^{-\lambda_{0}-\lambda_{1}x-\ldots -\lambda_{m}x^{m}}$$

where Lagrange multipliers $\lambda_{0},\ldots ,\lambda_{m}$ satisfy the following relations:

(5) $$\overset{1}{\underset{0}{\int }}\exp\left(-\overset{m}{\underset{j\;=1}{\sum }}\lambda_{j}x^{j}\right)\mathrm{dx}=\exp\left(\lambda_{0}\right)$$

(6) $$\frac{\int_{0}^{1}x^{i}\exp\left(-\sum_{j=1}^{m}\lambda_{j}x^{j}\right)\mathrm{dx}}{\int_{0}^{1}\exp\left(-\sum_{j=1}^{m}\lambda_{j}x^{j}\right)\mathrm{dx}}=\mu_{i},\;\;\;i=1,\ldots ,m$$

From a practical point of view, determining the maximum entropy of a pdf from given moments comes down to solving a nonlinear system of m Equation (6), creating a challenging nonlinear numerical problem faced by unbalanced nonlinearities, numerical instability and lack of convergence, especially for larger numbers of moments.

## 3. Numerical Algorithms Using Fup Basis Functions

This chapter describes two basic numerical algorithms for estimating the probability density function from a finite number of known moments. Both algorithms for solving the classical moment problem were developed using atomic basis functions, one of the algebraic type, ${\mathrm{Fup}}_{4}\left(x\right)$, and one of the exponential type, ${\mathrm{EFup}}_{4}\left(x,\omega \right)$.

**Figure 1:**
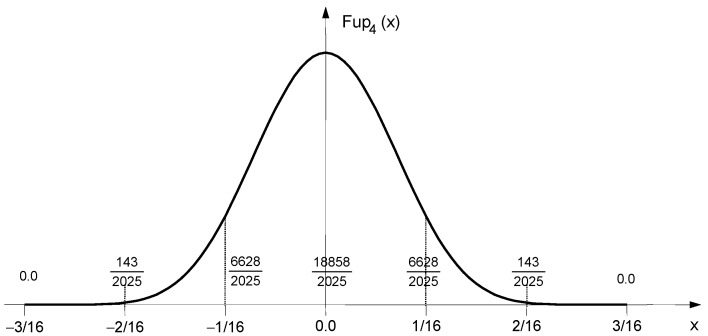
Function ${\mathrm{Fup}}_{4}\left(x\right)$.

The function ${\mathrm{Fup}}_{4}\left(x\right)$ is shown in Figure 1.

**Figure 2:**
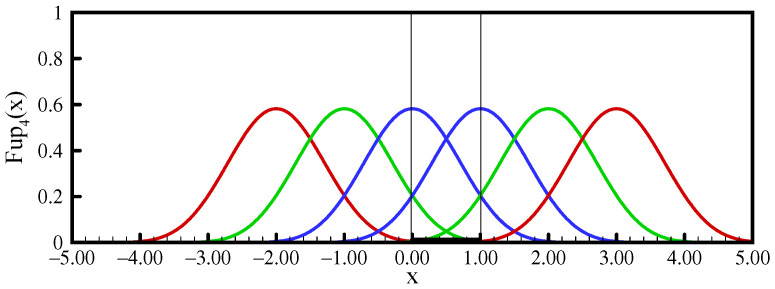
Linear combination of the ${\mathrm{Fup}}_{4}\left(x\right)$ functions over the [0,1] interval.

The function support contains six characteristic intervals. By a linear combination of these functions, shifted from each other by the length of the characteristic interval, algebraic polynomials up to the fourth degree can be accurately represented (see Appendix A, Appendix A.1). To approximate the pdf using the maximum entropy principle in the range [0,1], a base of at least six basis functions, ${\mathrm{Fup}}_{4}\left(x\right)$, is required, as shown in Figure 2.

**Figure 3:**
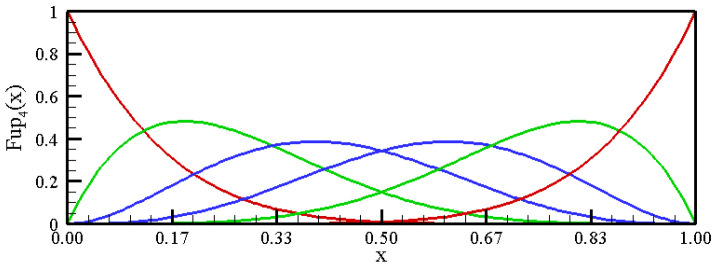
Linear combination of the modified ${\mathrm{Fup}}_{4}\left(x\right)$ functions over the [0,1] interval.

Therefore, the MaxEnt optimization procedure for estimating the probability density function starts from the sixth moment (with the zeroth moment engaged, i.e., m=5). For numerical operations in the domain to be performed efficiently, it is necessary to modify basis functions that have non-zero values within the domain and the vertices of which are outside the domain by expressing them in the form of a linear combination of the original basis functions [50]. Figure 3 shows the basis functions in the observed domain for the estimation with six moments (m=5).

**Figure 4:**
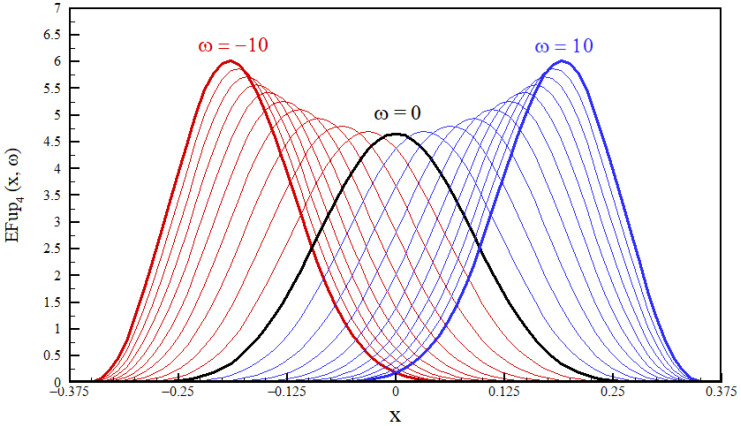
Function ${\mathrm{EFup}}_{4}\left(x,\omega \right)$ for −10≤ω≤10.

Fup basis functions of the exponential type are much less well-investigated than those of the algebraic type [56]. The linear combination of the functions ${\mathrm{EFup}}_{n}\left(x,\omega \right)$ can accurately represent the exponential function $E\left(x\right)=e^{2^{n}\omega x}$ (Appendix A, Appendix A.2). The ${\mathrm{EFup}}_{4}\left(x,\omega \right)$ function retains all the properties of the ${\mathrm{Fup}}_{4}\left(x\right)$ function, such as smoothness, finiteness, polynomial development, etc. The special feature of these functions is that they contain the parameter ω, which allows them to change the slope depending on the parameter value, as shown in Figure 4.

Figure 4 shows function ${\mathrm{EFup}}_{4}\left(x,\omega \right)$ for different values of parameter ω. It can be seen that the function ${\mathrm{EFup}}_{4}\left(x,\omega \right)$ for ω>0 is tilted to the right, and if ω<0, the function is tilted to the left. In the boundary case when ω=0, the function is symmetric and identical to the algebraic function ${\mathrm{Fup}}_{4}\left(x\right)$. Thus, a vector function space of the functions ${\mathrm{EFup}}_{n}\left(x,\omega \right)$ also contains functions ${\mathrm{Fup}}_{n}\left(x\right)$.

This property of EFup functions of the exponential type gives them an advantage over other finite functions (such as algebraic Fups, splines, wavelets), and it is their flexibility that allows them to “adapt” more to the solution. On a similar track, exponential splines that also contain a tension parameter are used in the literature; these are often applied to solve the singularly perturbed boundary problem [58,59]. When using the exponential type of atomic basis functions, the basic task is to determine the value of the parameter ω. Since the optimization problem of maximum entropy is the search for a pdf that maximizes the Shannon integral (3), the above will be taken as the criterion for choosing the parameter ω. Thus, of all possible solutions obtained for different values of the parameter ω, the one that gives the maximum value of the Shannon entropy functional (3) is adopted.

### 3.1. Classic MaxEnt Algorithm (Algorithm 1)

Here, we describe the procedure for solving the problem of pdf estimation from known moments using the principle of maximum entropy and ${\mathrm{Fup}}_{4}\left(x\right)$ basis functions [34]. The procedure is simplified in relation to [34] and consists of the following steps:

1. Let the set of m algebraic moments, $\mu_{i}$, be given on the general interval [a,b]. Its values on the unit interval [0,1] are calculated using a simple linear transformation.
2. In expression (4), we express the monomials $x^{i},\;i=0,\ldots ,m$ as a linear combination of basis functions ${\mathrm{Fup}}_{4}{}_{j}\left(x\right),\;j=0,\ldots ,m$, as shown in Appendix A, Appendix A.1.
  Since the ${\mathrm{Fup}}_{n}$ basis functions exactly describe algebraic monomials up to and including the nth order in the algorithm from [34], the residual function $\varepsilon_{i}\left(x\right)$ is introduced, i.e.,:
  (7) $$x^{i}=\overset{m}{\underset{j\;=0}{\sum }}d_{\mathrm{ij}}{\mathrm{Fup}}_{n}{}_{j}\left(x\right)+\varepsilon_{i}\left(x\right),\;\;i=0,\ldots ,m$$
  where
  (8) $$\begin{array}{cc} \varepsilon_{i}\left(x\right)\equiv 0 & \mathrm{for}\;i\leq n\\ \varepsilon_{i}\left(x\right)\neq 0 & \mathrm{for}\;i>n \end{array}$$
  are residual functions that describe the difference between monomials of the mth order and their Fup_n_ approximation, and $d_{\mathrm{ij}}$ presents the connection matrix, which depends on the Fup_n_ approximation and on the location and number of basis functions and, consequently, moments. For instance, the collocation procedure [48] calculates exact monomials in the collocation points, and residual functions $\varepsilon_{i}\left(x\right)$ fluctuate around zero, with significantly smaller values than Fup basis functions. For increasing numbers of moments and basis functions, the residual functions $\varepsilon_{i}\left(x\right)$ converge to zero, i.e., in the limit, for m→∞, $\varepsilon_{i}\left(x\right)$ is zero. Given the fact that the influence of lower moments in the process of determining the pdf is the largest, in this paper, we choose the Fup basis function of the higher order, i.e., n=4, which allows us to simplify the algorithm from [34] (leaving in mind that monomials above the fourth degree are now expressed approximately):
  (9) $$x^{i}\approx \overset{m}{\underset{j\;=0}{\sum }}d_{\mathrm{ij}}{\mathrm{Fup}}_{4}{}_{j}\left(x\right),\;\;i=0,\ldots ,m$$
  Next, we calculate the connection matrix $d_{\mathrm{ij}}$. Unlike [34], where the connection matrix $d_{\mathrm{ij}}$ is obtained by the collocation method, here, it is obtained by the Galerkin method:
  (10) $$\begin{array}{c} \overset{m}{\underset{j\;=0}{\sum }}d_{\mathrm{ij}}\overset{1}{\underset{0}{\int }}{\mathrm{Fup}}_{4}{}_{j}\left(x\right){\;\mathrm{Fup}}_{4}{}_{k}\left(x\right)\mathrm{dx}=\overset{1}{\underset{0}{\int }}x^{i}{\;\mathrm{Fup}}_{4}{}_{k}\left(x\right),\;\;i=0,\ldots ,m,\\ \;k=0,\ldots ,m \end{array}$$
  Multiplying expression (9) with the optimal pdf in the form
  (11) $$f^{*}\left(x\right)=e^{\sum_{j\;=0}^{m}\gamma_{j}{\mathrm{Fup}}_{4}{}_{j}\left(x\right)}$$
  and integrating over the domain [0,1], we can relate the classical algebraic and ${\mathrm{Fup}}_{4}$ moments
  (12) $$\mu_{i}=\overset{m}{\underset{j\;=0}{\sum }}d_{\mathrm{ij}}\cdot \mu_{j}{}^{{\mathrm{Fup}}_{4}}\;;i=0,\ldots ,m$$
  where $\mu_{i}$ represents the given moments of the requested pdf, while
  (13) $$\mu_{i}{}^{{\mathrm{Fup}}_{4}}=\overset{1}{\underset{0}{\int }}{\mathrm{Fup}}_{4}{}_{i}\left(x\right)\cdot e^{\sum_{j\;=0}^{m}\gamma_{j}{\mathrm{Fup}}_{4}{}_{j}\left(x\right)}\mathrm{dx}$$
  represents the moments of the ${\mathrm{Fup}}_{4}$ basis functions. Since the Lagrange multipliers, $\gamma_{j}$, are unknown, the system of Equation (13) must be solved iteratively.
3. The algorithm starts with the initial pdf assumption, i.e., for the step k=0, the initial values of the Lagrange multipliers $\gamma_{j}{}^{\left(k\;=0\right)},\;j=0,\ldots ,m$ are selected, from which the initial pdf is calculated according to (11).
  For simplicity, for initial values, $\gamma_{j}{}^{\left(k\;=0\right)},$ we take zero values, as the numerical procedure is not sensitive to the selection of the initial pdf. Then, the Fup moments $\mu_{j}{}^{{\mathrm{Fup}}_{4}}\;;j=0,\ldots ,m$ are determined in the kth iterative step by solving system (12).
4. From the known Fup moments, the problem of pdf estimation using the principle of maximum entropy is now solved:
  (14) $$\overset{1}{\underset{0}{\int }}{\mathrm{Fup}}_{4}{}_{i}\left(x\right)\cdot e^{\sum_{j=0}^{m}\gamma_{j}{\mathrm{Fup}}_{4}{}_{j}\left(x\right)}\mathrm{dx}=\mu_{i}{}^{{\mathrm{Fup}}_{4}}\;;\;i=0,\ldots ,m$$
  To determine the Lagrange multipliers, $\gamma_{j}$, the nonlinear system of Equation (14) is not solved at once but is solved iteratively by the classical Newton-Raphson method, equation by equation, using Romberg’s numerical integration.
5. The index of the current equation, i, is set. The correction of the ith Lagrange coefficient is performed using the classical Newton iterative procedure. If we demand that the residual function of the Lagrange coefficient, $\Psi \left(\gamma_{i}^{k}\right)\;$, weighs zero, then for the k+1 iterative step, the relation holds:
  (15) $$\gamma_{i}^{k+1}=\gamma_{i}^{k}-\frac{\Psi \left(\gamma_{i}^{k}\right)}{\;\Psi \prime \left(\gamma_{i}^{k}\right)}$$
  In the current iterative step, k, the correction of the value $\gamma_{i}^{k}$ is required in a way so that first the residual of the Lagrange coefficient belonging to a particular moment is determined:
  (16) $$\Psi \left(\gamma_{i}^{k}\right)=\overset{1}{\underset{0}{\int }}{\mathrm{Fup}}_{4}{}_{i}\left(x\right)\cdot e^{\sum_{j\;=0}^{m}\gamma_{j}{\mathrm{Fup}}_{4}{}_{j}\left(x\right)}\mathrm{dx}-\mu_{i}{}^{{\mathrm{Fup}}_{4}}$$
  and then the first derivation of that residual:
  (17) $$\;\Psi \prime \left(\gamma_{i}^{k}\right)=\overset{1}{\underset{0}{\int }}{\mathrm{Fup}}_{4}{}_{i}\left(x\right)\cdot e^{\sum_{j\;=0}^{m}\gamma_{j}{\mathrm{Fup}}_{4}{}_{j}\left(x\right)}\cdot {\mathrm{Fup}}_{4}{}_{i}\left(x\right)\;\mathrm{dx}$$
  and the correction of the Lagrange multiplier in the kth iterative step:
  (18) $${\Delta \gamma }_{i}^{k}=-\frac{\Psi \left(\gamma_{i}^{k}\right)}{\;\Psi \prime \left(\gamma_{i}^{k}\right)},\;{\;\gamma }_{i}^{k+1}=\gamma_{i}^{k}+\Delta \gamma_{i}^{k}$$

If all values of $\Delta \gamma_{i}^{k},\;i=0,\ldots ,m$ are less than the given tolerance, the iterative procedure is stopped; otherwise, step k+1 is taken and point five is repeated until the convergence condition is satisfied or until the maximum number of given iterative steps is reached.

### 3.2. Fast Linear Algorithm (Algorithm 2)

Instead of solving a nonlinear system of Equation (13) in the classical MaxEnt problem, which, in some cases, can cause greater numerical instabilities and slow the solution convergence, the process of pdf estimation from known moments using the maximum entropy principle can be simplified by reducing the problem to solving the linear system of equations. The procedure is described using ${\mathrm{EFup}}_{4}\left(x,\omega \right)$ basis functions and consists of the following steps:

1. The probability density function is assumed in the form of a linear combination of ${\mathrm{EFup}}_{4}$ basis functions:
  (19) $$f\left(x\right)\approx \overset{m}{\underset{j\;=0}{\sum }}c_{j}\cdot {\mathrm{EFup}}_{4}{}_{j}\left(x,\omega \right)$$
  The procedure of pdf determination from the given moments, $\mu_{i}$, comes down to directly solving a linear system of equations:
  (20) $$c_{j}\overset{1}{\underset{0}{\int }}x^{i}\cdot {\mathrm{EFup}}_{4}{}_{j}\left(x,\omega \right)\mathrm{dx}=\mu_{i}\;;\;i,j=0,\ldots ,m$$
  from which follow the coefficients of the linear combination, $c_{j}$.
2. By including the calculated coefficients, $c_{j}$, in expression (19), a pdf estimation is obtained, which can also have negative values. Since the probability density function must be positive by definition, i.e., non-negative in the range [0,1], the corresponding R-function [60] is used to remove the negative part of the function (or to control the lower limit of the pdf):
  (21) $$f^{*}\left(x\right)=\frac{1}{1+\alpha }\left(f_{1}\left(x\right)+f_{2}\left(x\right)+\sqrt{f_{1}{\left(x\right)}^{2}+f_{2}{\left(x\right)}^{2}-2\alpha \cdot f_{1}\left(x\right)\cdot f_{2}\left(x\right)}\right)$$
  where $\alpha =\alpha \left(f_{1}\left(x\right),f_{2}\left(x\right)\right)$ is an arbitrary function that satisfies the condition:
  (22) $$-1<\alpha \left(f_{1}\left(x\right),f_{2}\left(x\right)\right)\leq 1$$
  For α=1 follows:
  (23) $$f^{*}\left(x\right)=\frac{1}{2}\left\{f_{1}\left(x\right)+f_{2}\left(x\right)+\left|f_{1}\left(x\right)-f_{2}\left(x\right)\right|\right\}$$
  In the case of maximum entropy, $f_{1}\left(x\right)$ represents the probability density function, and the function $f_{2}\left(x\right)$ is the lower limit that has a value of zero, so (21) becomes:
  (24) $$f^{*}\left(x\right)=\frac{1}{2}\left\{f_{1}\left(x\right)+\left|f_{1}\left(x\right)\right|\right\}$$
  If f(x) obtained from (19) i (20) is already a non-negative function, then (21) has no effect on the solution of the pdf.
3. The function obtained according to expression (21) is normalized for the pdf to fulfill the basic probability condition (1). The moments of the pdf obtained in this way are calculated, compared with the given moments and the error of the calculated moments is estimated. The value of Shannon entropy is also calculated according to expression (3).
4. The procedure is repeated for a number of real parameters, ω, within the selected interval. The interval can be arbitrary and contain positive and negative values of parameters. When ω=0, the ${\mathrm{EFup}}_{4}$ basis functions are identical to the ${\mathrm{Fup}}_{4}$ basis functions.
5. A pdf with the parameter ω that gives the highest value of Shannon entropy according to expression (3) is selected.

In the limit, when m→∞, the obtained function f(x) approaches the exact pdf function because it satisfies all moments. For a finite number of moments, the procedure is approximate, but the possibility of choosing the parameter, ω, which gives the maximum possible value of the functional (3) and also adapts to the shape of the pdf, often allows for a very good approximation of the pdf. On the other hand, the pdf vector space of the maximum entropy (4) shows that it belongs to a function of the exponential type; thus, the ${\mathrm{EFup}}_{4}$ basis functions make very good candidates for the linear approximation (19). The procedure is particularly suitable for greater numbers of moments when Algorithm 1 can fail.

## 4. Examples

In this section, the approximation possibilities of the proposed algorithms and their computational efficiency are explored through various examples of pdfs in order to show the advantages and disadvantages of nonlinear classical Algorithm 1 and linear Algorithm 2.

### 4.1. Example 1–Impulse Function

The efficiency of Algorithm 1 is demonstrated in the example of a numerically demanding probability density function, which, for the MaxEnt moment problem (1–2), represents a challenging numerical problem. It is an impulse function that is very similar to Dirac’s function in the form:

(25) $$\delta \left(x\right)=\frac{256}{\sqrt{\pi }\cdot \mathrm{ERF}\left(128\right)}e^{-4096{\left(4x-2\right)}^{2}}$$

**Figure 5:**
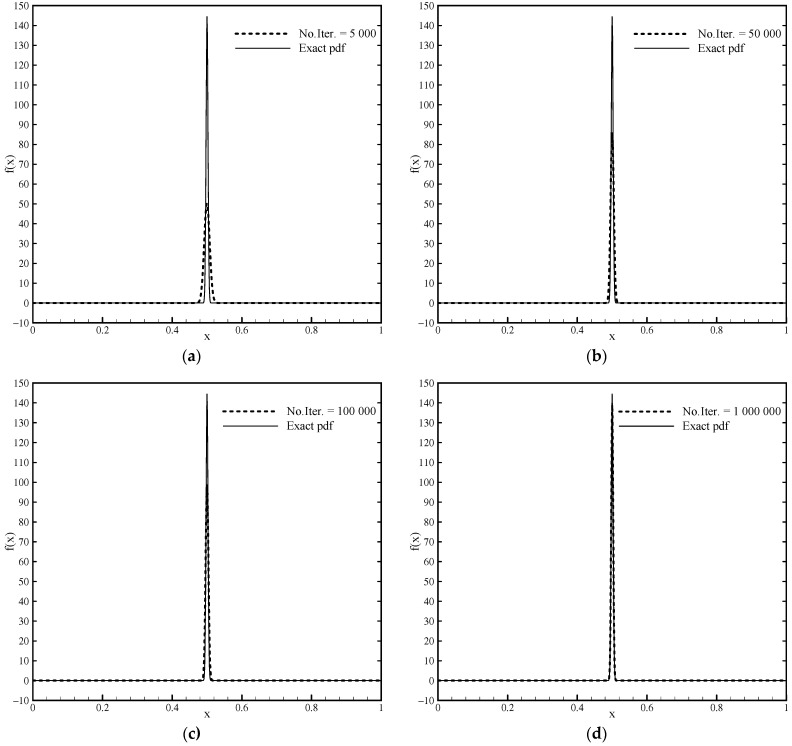
Approximation of the impulse pdf function for m=5 in the nonlinear procedure of Algorithm 1 with different numbers of iterations: (**a**) No.Iter. = 5000, (**b**) No.Iter. = 50,000, (**c**) No.Iter. = 100,000, (**d**) No.Iter. = 1,000,000.

The peak of this function is in the coordinate x=0.5 with a value of 144.4325. Since this function is symmetric, the parameter ω is zero, which means that Algorithm 1 and basis functions of the algebraic type ${\mathrm{Fup}}_{4}\left(x\right)$ are used. It is interesting to show the convergence of the solution (Figure 5) that is obtained with only six basis functions, i.e., using the minimum number of moments for this algorithm, with an increase in the number of iterative nonlinear steps.

For $3\cdot 10^{6}$ iterations, the achieved pdf shape practically coincides with the shape of the analytical function. Table 1 shows how the moment error calculated using Algorithm 1 decreases with the total number of iterations and how the value of the pdf increases at the point x=0.5 of the observed area.

This example shows the accuracy and stability of numerical Algorithm 1 but also the deficiency of a nonlinear algorithm that requires a large number of iterations. With only six moments, an excellent solution is achieved. With a fast linear Algorithm 2 for this example of a symmetric impulse pdf function, it is not possible to achieve a satisfactory solution, nor can one be achieved with a larger number of moments due to, among other things, the oscillations that occur around the point x=0.5. For example, the maximum pdf value obtained with 20 moments is 16.097, which is very far from the exact value.

### 4.2. Example 2—Bimodal Function

**Figure 6:**
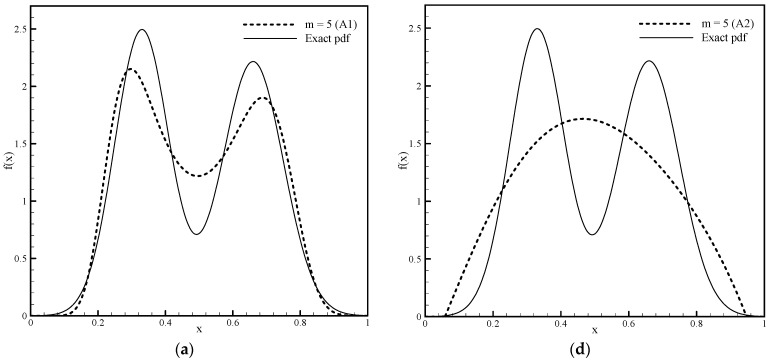

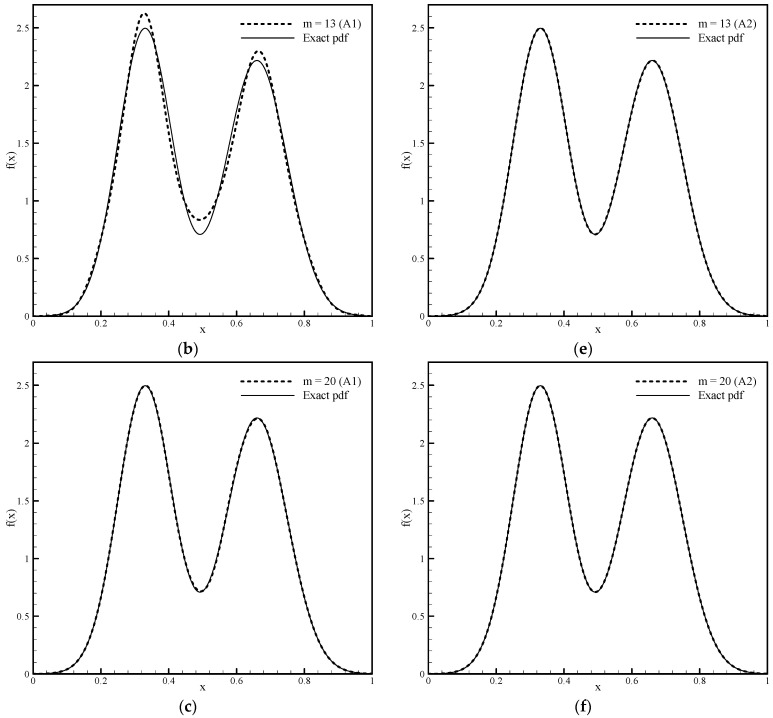
Approximation of the bimodal pdf function using Algorithm 1 with given (**a**) 6, (**b**) 14 and (**c**) 21 moments and approximation of the same using Algorithm 2 with given (**d**) 6, (**e**) 14, (**f**) and 21 moments.

In the second example, we consider a bimodal function composed of two Gaussian distributions normalized to the domain [0,1]. Figure 6a–c show approximations of the bimodal pdf obtained by Algorithm 1, and Figure 6d–f show approximations obtained using Algorithm 2 for m=5, 13 and 20. Since this function is approximately symmetric, a value close to zero is obtained for the parameter ω so that the numerical calculation is done using ${\mathrm{Fup}}_{4}\left(x\right)$ basis functions.

Improving the pdf estimation by increasing the number of moments taken into account, besides its shape, is monitored through the achieved accuracy of the moments and through the values of the Shannon entropy functional using expression (3). The exact value of the Shannon integral for a bimodal function is H(f)=−0.42420.

Table 2 and Table 3 show that as the number of constraints of the required pdf function increases, the absolute moment error decreases, while the approximation of the Shannon entropy functional (3) improves for calculations using Algorithm 1 and Algorithm 2, respectively. Algorithm 1 approximately satisfies the moments due to solving a nonlinear system of equations and the selected accuracy criterion (“threshold”), as well as approximation of monomials with Fup_4_ basis functions. On the other hand, functional (3) is also approximately calculated, and with an increasing number of moments, converges relatively slowly to the exact value of H(f)=0.42420. Algorithm 2 only solves a linear system that satisfies the given moments. With a small number of moments (m=5) in the symmetric type of pdf, the solution is significantly worse than that of Algorithm 1, which is reflected not only in the visual effect but also in the accuracy of the moments and the value of the functional (3). Yet, when the number of moments reaches m=13, very accurate results are obtained with a much simpler procedure.

**Figure 7:**
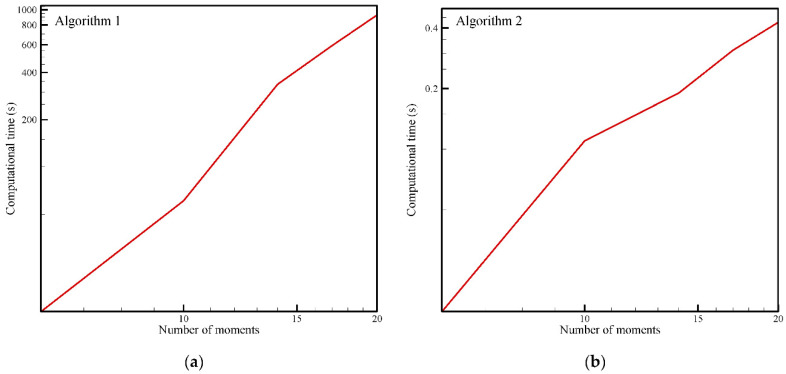
Required calculation time (“CPU time”) as a function of the number of moments for: (**a**) Algorithm 1, (**b**) Algorithm 2.

The calculation time (“CPU time”) depending on the number of moments taken for both algorithms is graphically shown in Figure 7. The computer time is given in seconds and is obtained using the classic programming language Fortran on a computer with an Intel i7 2.80 GHz processor. As the number of moments increases, both algorithms require a nonlinear increase in computation time. When the number of moments exceeds the limit of m=10−12 a significant saving of computation time is obtained with Algorithm 2. As seen in Figure 7, in the log-log scale, an approximately linear dependence of the number of moments and CPU time is obtained.

From the presented results, it can be concluded that the classical nonlinear MaxEnt Algorithm 1 gives an approximate pdf shape with only six moments. By increasing the number of moments or the number of basis functions, the calculated pdf shape approaches the exact form of the bimodal function, the accuracy of the moments increases and the value of the Shannon entropy functional also approaches the exact value. It can be said that a satisfactory solution is achieved with 21 moments.

However, due to the large number of iterative steps required to solve a nonlinear system of equations, this procedure is a computationally expensive and also very numerically sensitive. Algorithm 2 is a fast algorithm, is not numerically demanding and the procedure is stable. The system of linear equations is solved directly, and, as can be seen in Figure 7, the required computational time is significantly less than that of Algorithm 1. Note that the pdf estimation for a small number of moments is not sufficient; with nine moments, the obtained pdf shape has the characteristics of the required solution that Algorithm 1 provides with only six moments.

However, for a larger number of moments (m≥13), the accuracy of the solution increases significantly (see Table 3) both in terms of pdf approximation approaching the exact function shape and in terms of the moment accuracy and the Shannon integral value (3). Using 21 moments, a pdf solution was achieved in which the absolute error of the moments was practically equal to zero and the value of H(f) coincided with the exact value. The advantages of both numerical algorithms for estimating the pdf from known moments can be combined in a way by first obtaining the shape of the required function with six moments using the Fup MaxEnt Algorithm 1, from which one can conclude the basic characteristics of the function, and then with the fast Algorithm 2 to get a more accurate pdf estimation using a greater number of moments.

### 4.3. Example 3—Beta Function

The third example of the beta distribution is given in the form:

(26) $$f\left(x\right)=3990x^{18}{\left(1-x\right)}^{2}$$

When applying atomic basis functions of exponential type ${\mathrm{EFup}}_{4}\left(x,\omega \right)$, the basic task is to determine the value of the parameter ω. Function (26) is asymmetric and inclined to the right, which means that the parameter ω>0 (see Figure 4). Since the principle of maximum entropy requires a pdf that maximizes the Shannon integral (3), this condition will be used as a criterion for selecting the parameter ω. The procedure is reduced to the simultaneous direct solution of the linear system (20) for different values of the parameter ω. Of all the numerical solutions thus obtained, the one that gives the maximum value of Shannon entropy (3) is adopted.

**Figure 8:**
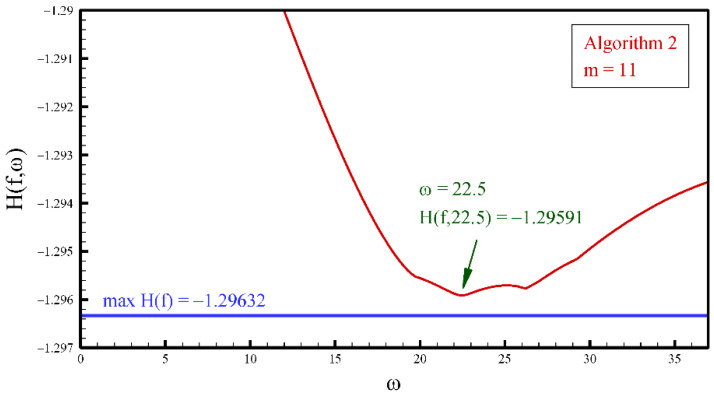
Shannon integral values (3) for beta pdf (26) and m=11 obtained with different values of the parameter ω.

The procedure for determining the parameter ω for pdf estimation by the principle of maximum entropy with 12 moments using basis functions ${\mathrm{EFup}}_{4}\left(x,\omega \right)$ is illustrated in Figure 8. The exact value of the integral (3) for the beta function of pdf (26) is H(f)=1.29632, and the closest value is obtained with ω=22.5.

**Figure 9:**
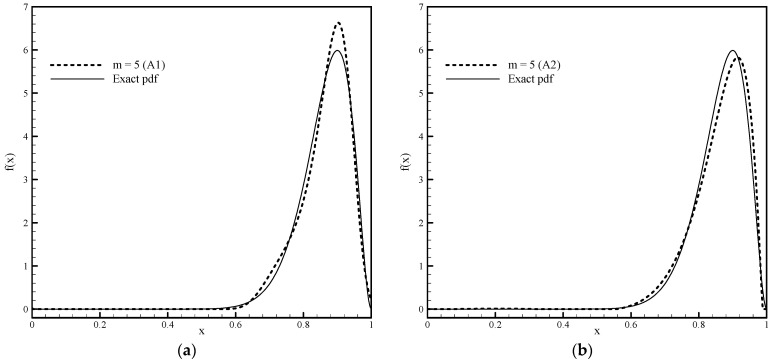
Estimates of pdf for beta distribution obtained for given six moments with (**a**) Algorithm 1 and (**b**) Algorithm 2.

Figure 9 compares the numerical solutions obtained by applying basis functions ${\mathrm{Fup}}_{4}\left(x\right)$ and ${\mathrm{EFup}}_{4}\left(x,\omega \right)$ with Algorithms 1 and 2, respectively, for m=5. The value of the parameter ω for Algorithm 2, determined by the previously described procedure for pdf estimation with six moments, is ω=23.0.

From Figure 9, we can see the advantage that exponential finite basis functions have in relation to basis functions of the algebraic type when it comes to pdf approximation, which is quite an asymmetric function. The greater accuracy of numerical solutions obtained using basis functions ${\mathrm{EFup}}_{4}\left(x,\omega \right)$ can also be seen in Table 4, where the absolute errors are given of approximate moments for calculations with both algorithms using algebraic and exponential Fup basis functions.

Table 4 shows the convergence of the values of the Shannon entropy functionals (3) obtained by increasing the number of moments starting from m=5 using Algorithms 1 and 2 and using the atomic basis functions ${\mathrm{Fup}}_{4}\left(x\right)$ and ${\mathrm{EFup}}_{4}\left(x,\omega \right)$. In this example of the probability density function, the advantage that exponential finite functions have over basis functions of the algebraic type comes down to expression. It can be seen that the functions ${\mathrm{EFup}}_{4}\left(x,\omega \right)$ achieve a significantly better convergence of the numerical values of the Shannon integral to the exact value of H(f)=−1.29632. Since exponential functions adapt better to the shape of the MaxEnt problem solution thanks to the parameter ω, they ensure the stability of the numerical procedure, which is especially important for the classical MaxEnt algorithm. In this example, this indicated an extreme numerical sensitivity when using ${\mathrm{Fup}}_{4}\left(x\right)$ basis functions.

For practical application, i.e., for estimating the pdf from known moments when the form of the function is unknown, the advantages of both described numerical algorithms can be used. First, using the nonlinear MaxEnt Algorithm 1 with basis functions ${\mathrm{Fup}}_{4}\left(x\right)$, which are easier to apply than ${\mathrm{FEup}}_{4}\left(x,\omega \right)$, we get the appearance of the required function with six moments (m=5), from which can be drawn certain conclusions about the basic characteristics of the pdf, such as its shape, position peaks, number of peaks and inclination, while the number of statistical moments required to accurately describe all the properties of the pdf can be assumed. If an approximately symmetric function is obtained, such a first approximation, the calculation can be continued with the basis functions ${\mathrm{Fup}}_{4}\left(x\right)$.

If the first approximation gives an asymmetric function or a larger number of moments is required, then using the fast linear Algorithm 2, the parameter ω is determined with the criterion for finding the maximum of the Shannon entropy functional (3), and the calculation continues with exponential functions ${\mathrm{FEup}}_{4}\left(x,\omega \right)$. If it is a simple pdf for which less than six moments are enough, Algorithm 1 with ${\mathrm{Fup}}_{2}$ [34] or ${\mathrm{Fup}}_{1}$ basis functions can be used. In this way, using the accuracy and robustness of the classic nonlinear MaxEnt Algorithm 1 up to 10–12 moments, we can solve a large number of problems and diagnose the condition if more moments are needed, especially with pdf asymmetry, when linear Algorithm 2 becomes much simpler and more efficient. With this hybrid technique, a large number of challenging problems can be solved in a much easier way than could be solved individually with the two presented algorithms.

## 5. Conclusions

In this paper, the application has been shown of numerical algorithms based on the maximum entropy principle using Fup basis functions that belong to the class of atomic basis functions with a compact support. The pdf examples selected in this paper demonstrate the efficiency of two algorithms for probability-density-function estimation from a finite number of known moments. These algorithms use Fup basis functions of the algebraic and exponential types. The simulation results show the convergence properties of the proposed algorithms by increasing the number of moments taken into account. The example of a pdf in the form of a pulse function shows the accuracy and stability of the numerical procedure of Algorithm 1, where with only six moments, a satisfactory solution can be achieved by increasing the number of iterations in the calculation.

With such demanding pdfs, the number of iterative steps in the nonlinear procedure becomes very large. The characteristics of numerical solutions obtained with Algorithms 1 and 2 using algebraic finite basis functions are shown in the example of a demanding bimodal function. The classic nonlinear MaxEnt Algorithm 1 gives a good pdf shape approximation with only six moments. By increasing the number of moments or the number of basis functions, the approximate solution converges to the exact function. However, the numerical procedure is computationally expensive and sensitive to the computer’s precision, so the optimization problem becomes poorly conditioned. Fast linear Algorithm 2 ensures complete stability of the procedure, but a higher number of moments is required to satisfy the accuracy of the pdf estimation.

The third example of a pdf is a beta function that represents a polynomial of the 20th degree, which is why it is numerically demanding to approximate. Nonlinear Algorithm 1 showed special sensitivity in this pdf when applying algebraic finite basis functions. In the example of quite an asymmetric pdf function, the advantage of exponential finite basis functions over functions of the algebraic type can be noted both in the stability of the nonlinear classical MaxEnt algorithm and in the achieved accuracy of calculated pdf moments and calculated Shannon integral values as a basic criterion for pdf estimation by the principle of maximum entropy.

For practical applications, i.e., for pdf estimation from known moments when the shape of the function is unknown, we can use the advantages of both numerical algorithms described here, which we defined as a hybrid strategy in this article.

## Figures and Tables

**Table 1:** Absolute moment errors and pdf values in relation to the total number of iterations for m=5.

Total Number of Iterations	5 × 10^3^	5 × 10^4^	10^5^	10^6^	3 × 10^6^
Absolute moment error	0.26 × 10^−2^	0.68 × 10^−3^	0.42 × 10^−3^	0.12 × 10^−4^	0.29 × 10^−7^
Maximum pdf value	50.154	85.738	99.018	138.902	144.279

**Table 2:** Absolute moment error and values of functional (3) for Algorithm 1.

Number of Moments	6 (m = 5)	14 (m = 13)	21 (m = 20)
Absolute moment error	0.33 × 10^−6^	0.36 × 10^−7^	0.47 × 10^−9^
Value of the Shannon functional	−0.39841	−0.42283	−0.42419

**Table 3:** Absolute error of moments and values of functional (3) for Algorithm 2.

Number of Moments	6 (m = 5)	14 (m = 13)	21 (m = 20)
Absolute moment error	0.14 × 10^−1^	0.18 × 10^−4^	0.25 × 10^−33^
Value of Shannon functional	−0.24727	−0.42416	−0.42420

**Table 4:** Absolute moment errors and Shannon integral values obtained for m=5, 11 and 20 for Algorithms 1 and 2 with ${\mathrm{Fup}}_{4}\left(x\right)$ basis functions and ${\mathrm{EFup}}_{4}\left(x,\omega \right)$, respectively.

No. of Moments		6 (m = 5)	12 (m = 11)	21 (m = 20)
Abs. Moment Error	Algorithm 1	0.69 × 10^−5^	0.120 × 10^−4^	0.743 × 10^−4^
	Algorithm 2	0.337 × 10^−2^	0.144 × 10^−4^	0.194 × 10^−6^
Shannon Integral	Algorithm 1	−1.29590	−1.29571	−1.29686
	Algorithm 2	−1.29223	−1.29591	−1.29631
ω	Algorithm 2	23.0	22.5	17.2

## Appendix A. Fup_n_ and EFup_n_ Basis Functions

### Appendix A.1. Calculation and Approximation Properties of the Fup_n_ Basis Functions

Atomic basis functions are infinitely-differentiable functions with compact support [35,56]. Atomic functions are defined as solutions of differential functional equations of the following type:

(A1) $$\mathrm{Ly}\left(x\right)=\lambda \overset{M}{\underset{k=1}{\sum }}C_{k}\;y\left(\mathrm{ax}-b_{k}\right)$$

where L is a linear differential operator with constant coefficients, λ is a nonzero scalar, $C_{k}$ are coefficients of the linear combination, a>1 is a parameter that defines the length of the compact support, and $b_{k}$ are coefficients that determine displacements of the basis functions. Rvačev and Rvačev, in their pioneering work [35], called these basis functions ‘‘atomic” because they span the vector spaces of all three fundamental functions in mathematics: algebraic, exponential and trigonometric polynomials.

The simplest function, which is the most studied of the atomic basis functions, is the up(x) function. The function up(x) is a smooth function with compact support over [−1,1], which is obtained as a solution of a differential functional equation

(A2) $${\mathrm{up}}^{\prime }\left(x\right)=2\cdot \mathrm{up}\left(2x+1\right)-2\cdot \mathrm{up}\left(2x-1\right)$$

with the normalized condition $\int_{-\infty }^{+\infty }\mathrm{up}\left(x\right)\mathrm{dx}=\int_{-1}^{1}\mathrm{up}\left(x\right)\mathrm{dx}=1$. The function up(x) can be expressed as an inverse Fourier transform:

(A3) $$\mathrm{up}\left(x\right)=\frac{1}{2\pi }\overset{+\infty }{\underset{-\infty }{\int }}e^{-\mathrm{itx}}\cdot \overset{\infty }{\underset{j\;=1}{\prod }}\frac{\sin\left(t/2^{j}\right)}{t/2^{j}}\mathrm{dt}$$

Since (A3) represents an exact but mathematically intractable expression, in [36,56], a more numerically adequate expression for calculating the function up(x) is provided:

(A4) $$\mathrm{up}\left(x\right)=1-\overset{\infty }{\underset{k=1}{\sum }}{\left(-1\right)}^{1+p_{1}+\ldots +p_{k}}{\;p}_{k}\overset{k}{\underset{j\;=0}{\sum }}C_{\mathrm{jk}}\cdot {\left(x-0,p_{1}\ldots {\;p}_{k}\right)}^{j}$$

where coefficients $C_{\mathrm{jk}}$ are rational numbers determined according to the following expression:

(A5) $$C_{\mathrm{jk}}=\frac{1}{j!}2^{j\left(j+1\right)/2}\mathrm{up}\left(-1+2^{-\left(k-j\right)}\right)\;;\;j=0\;,\;1\;,\;\ldots \;,\;k\;;\;k=1\;,\;2\;,\;\ldots \;,\;\infty$$

Calculation of $\mathrm{up}\left(-1+2^{-r}\right)$ r[0,∞] in binary-rational points (A5), as well as all details regarding the calculation of the function up(x) values, is provided in [56,61]. The argument $\left(x-0,p_{1}\ldots {\;p}_{k}\right)$ in (A1.4) is the difference between the real value of coordinate x and its binary form in k bits, where $p_{1}\ldots {\;p}_{k}$ are digits, 0 or 1, of the binary representation of the x coordinate. Therefore, the accuracy of the x coordinate computation, and thus the accuracy of the up(x) function at an arbitrary point, depends on machine accuracy.

The ${\mathrm{Fup}}_{n}\left(x\right)$ function satisfies the following differential-functional equation:

(A6) $${\mathrm{Fup}}_{n}^{\prime }\left(x\right)=2\overset{n+2}{\underset{k=0}{\sum }}\left(C_{n+1}^{k}-C_{n+1}^{k-1}\right)\cdot {\mathrm{Fup}}_{n}\left(2x-\frac{k}{2^{n+1}}+\frac{n+2}{2^{n+2}}\right)$$

where $C_{n+1}^{k}$ and $C_{n+1}^{k-1}$ are binomial coefficients and n is the Fup order. Index n also denotes the highest degree of the polynomial that can be expressed exactly as a linear combination of n+2 ${\mathrm{Fup}}_{n}\left(x\right)$ basis functions, uniformly displaced by a characteristic interval $2^{-n}$.

For n=0, ${\mathrm{Fup}}_{0}\left(x\right)=\mathrm{up}\left(x\right)$ since ${\mathrm{Fup}}_{n}\left(x\right)$ and its derivatives can be calculated using a linear combination of displaced up(x) functions:

(A7) $${\mathrm{Fup}}_{n}\left(x\right)=\overset{\infty }{\underset{k=0}{\sum }}C_{k}\left(n\right)\cdot \mathrm{up}\left(x-1-\frac{k}{2^{n}}+\frac{n+2}{2^{n+1}}\right)$$

where $C_{0}=2^{C_{n+1}^{2}}=2^{n\left(n+1\right)/2}$. In turn, $C_{k}\left(n\right)=C_{0}\left(n\right)\cdot C_{k}^{\prime }\left(n\right)$, where a recursive formula is used for calculating auxiliary coefficients $C_{k}^{\prime }\left(n\right)$:

(A8) $$\begin{array}{l} C_{0}^{\prime }\left(n\right)=1\;,\;\mathrm{when}\;k=0\;;\;i.e.,\;\mathrm{when}\;k>0\\ C_{k}^{\prime }\left(n\right)={\left(-1\right)}^{k}C_{n+1}^{k}-\sum_{j\;=1}^{\min\;\left\{\;k\;;\;2^{n+1}-1\right\}}{\;C}_{k-j}^{\prime }\left(n\right)\cdot \delta_{j+1} \end{array}$$

${\mathrm{Fup}}_{n}\left(x\right)$ is defined over the compact support $\left[-\left(n+2\right)\cdot 2^{-n-1},\;\left(n+2\right)\cdot 2^{-n-1}\right]$. Figure 1 in the manuscript shows the ${\mathrm{Fup}}_{4}\left(x\right)$ function.

Linear combination of displaced ${\mathrm{Fup}}_{n}\left(x\right)$ functions in the form:

(A9) $$\varphi \left(x\right)=\overset{\infty }{\underset{k=-\infty }{\sum }}D_{k}\cdot {\mathrm{Fup}}_{n}\left(x-\frac{k}{2^{n}}\right)$$

is a polynomial of nth degree if coefficients $D_{k}$ are the polynomials of degree n of index k. Coefficients $D_{k}$ are determined using collocation procedure [36].

Therefore, a polynomial of nth degree can be exactly expressed on a $2^{-n}$ long interval as a linear combination of basis functions obtained by function ${\mathrm{Fup}}_{n}\left(x\right)$ displacement over a characteristic interval $\Delta x_{n}=2^{-n}$.

A linear combination of displaced ${\mathrm{Fup}}_{4}\left(x\right)$ basis functions at the interval [0, 1] describes exactly the monomials:

(A10) $$\begin{array}{l} x^{0}=\frac{1}{2^{4}}\sum_{k=-2}^{3}{\mathrm{Fup}}_{4}\left(x-k/16\right)\\ x^{1}=\frac{\Delta x}{2^{8}}\overset{3}{\underset{k=-2}{\sum }}k\cdot {\mathrm{Fup}}_{4}\left(x-k/16\right)\\ x^{2}=\frac{{\left(\Delta x\right)}^{2}}{2^{12}}\sum_{k=-2}^{3}\left(k^{2}-\frac{4}{9}\right)\cdot {\mathrm{Fup}}_{4}\left(x-k/16\right)\\ x^{3}=\frac{{\left(\Delta x\right)}^{3}}{2^{16}}\sum_{k=-2}^{3}\left(k^{3}-\frac{4}{3}k\right)\cdot {\;\mathrm{Fup}}_{4}\;\left(x-k/16\right)\\ x^{4}=\frac{{\left(\Delta x\right)}^{4}}{2^{20}}\sum_{k=-2}^{3}\left(k^{4}-\frac{8}{3}k^{2}+\frac{857}{1350}\right)\cdot {\;\mathrm{Fup}}_{4}\;\left(x-k/16\right) \end{array}$$

Other higher-order monomials are expressed approximately. Differences between monomials and their ${\mathrm{Fup}}_{4}\left(x\right)$ approximations are defined by residual functions $\varepsilon_{i}\left(x\right)$ (see article, Point 3.1).

### Appendix A.2. Calculation and Approximation Properties of the EFup_n_ Basis Functions

The ${\mathrm{EFup}}_{n}\left(x,\omega \right)$ functions are atomic finite functions of class $C^{\infty }$ with compact support [35] and are the elements of the linear vector space ${\mathrm{EUP}}_{n}$. The simplest function from the class ${\mathrm{EFup}}_{n}\left(x,\omega \right)$ is the function Eup(x,ω) for n=0 [57], which, analogous to the algebraic ABF [56], is called the maternal function. The functions ${\mathrm{EFup}}_{n}\left(x,\omega \right)$ retain all the properties of ‘their’ maternal basis function, Eup(x,ω) [57]. The index ′n′ denotes the largest degree of an exponential polynomial that can be represented exactly in the form of a linear combination of shifted ${\mathrm{EFup}}_{n}\left(x,\omega \right)$ functions on a segment of length ${\Delta x}_{n}=2^{-n}$. The parameter ω gives them an additional property of ‘adaptability’ with respect to the ABF of the algebraic type (see Figure 4).

The Fourier transform (FT) of atomic basis functions ${\mathrm{EFup}}_{n}\left(x,\omega \right)$ is of the form:

(A11) $$F_{n}\left(t\right)=\overset{n+1}{\underset{j=1}{\prod }}\frac{\omega }{2^{j}\mathrm{sh}\left(\omega /2^{j}\right)}\cdot \frac{\mathrm{sh}\left(\omega /2^{j}+i\cdot t/2^{n+1}\right)}{\omega /2^{j}+i\cdot t/2^{n+1}}\cdot \overset{\infty }{\underset{k=n+2}{\prod }}\frac{\omega }{2\mathrm{sh}\left(\omega /2\right)}\cdot \frac{\mathrm{sh}\left(\omega /2+i\cdot t/2^{k}\right)}{\omega /2+i\cdot t/2^{k}}$$

and, according to expression (A11), is equal to the multiplication of (n+1) FT of exponential splines of zero degree $\varphi_{0}^{j}\left(x,\omega \right),$ normalized to the interval $h_{0}=2^{-n}$:

$$\begin{array}{c} \varphi_{0}^{j}\left(x,\omega \right)=\frac{2^{j-1}\cdot \omega }{\mathrm{sh}\left(\omega /2^{n-j+1}\right)}e^{2^{j}\omega x}\;,\;\;j=0,\ldots ,n\\ \mathrm{supp}\;\varphi_{0}^{j}\left(x,\omega \right)=\left[-2^{-\left(n+1\right)},2^{-\left(n+1\right)}\right] \end{array}$$

and the FT of maternal function Eup(x,ω) also compressed to the support $\mathrm{supp}\;\mathrm{Eup}\left(x,\omega \right)=\left[-2^{-\left(n+1\right)},2^{-\left(n+1\right)}\right]$. Thus, the function ${\mathrm{EFup}}_{n}\left(x,\omega \right)$ can be written using convolution theorem in the following form:

(A12) $$\begin{array}{l} {\mathrm{EFup}}_{n}\left(x,\omega \right)=\varphi_{0}^{0}\left(x,\omega \right)*\ldots {\;*\varphi }_{0}^{n}\left(x,\omega \right)\;*\;2^{n+1}\mathrm{Eup}\left(2^{n+1}x,\omega \right) \end{array}$$

The support of the function ${\mathrm{EFup}}_{n}\left(x,\omega \right)$ is an interval composed of (n+2) subintervals of length $2^{-n}$:

$${\mathrm{supp}\;\mathrm{EFup}}_{n}\left(x,\omega \right)=\left[-\left(n+2\right)\cdot 2^{-n-1},\;\left(n+2\right)\cdot 2^{-n-1}\right]$$

The characteristic points are the boundary points of the subintervals.

The inverse Fourier transform, i.e., the function ${\mathrm{EFup}}_{n}\left(x,\omega \right)$, with the satisfaction of the Paley-Wiener normalization condition, can be expressed in the form:

(A13) $${\mathrm{EFup}}_{n}\left(x,\omega \right)=\frac{1}{2\pi }\overset{\infty }{\underset{-\infty }{\int }}{\;e}^{-\mathrm{itx}}{\;F}_{n}\left(t\right)\;\mathrm{dt}$$

By developing expression (A13) in the Fourier series, the ˝original˝ of the function ${\mathrm{EFup}}_{n}\left(x,\omega \right)$ can be determined at arbitrary points. However, practically, the most optimal possibility of constructing functions ${\mathrm{EFup}}_{n}\left(x,\omega \right)$ is in the form of a linear combination of mutually shifted Eup(x,ω) basis functions.

(A14) $${\mathrm{EFup}}_{n}\left(x,\omega \right)=\overset{\infty }{\underset{k=0}{\sum }}C_{k}\left(n\right)\cdot \mathrm{Eup}\left(x-1-\frac{k}{2^{n}}+\frac{n+2}{2^{n+1}},\omega \right)$$

where $C_{k}\left(n\right)$ are the coefficients of the linear combination

(A15) $$C_{k}\left(n\right)=\frac{{\mathrm{EFup}}_{n}\left(-\frac{n+2}{2^{n+1}}+\frac{k+1}{2^{n}},\omega \right)-\sum_{i\;=1}^{k}C_{i-1}\left(n\right)\cdot \mathrm{Eup}\left(-1+\frac{k+2-i}{2^{n}},\omega \right)}{\mathrm{Eup}\left(-1+2^{-n},\omega \right)}$$

and the coefficients $C_{0}\left(n\right)$ are determined by

(A16) $$C_{0}\left(n\right)=\overset{n}{\underset{i\;=1}{\prod }}{\left(e^{\omega /2^{n-i+1}}+1\right)}^{i}$$

The derivatives and integrals of the functions ${\mathrm{EFup}}_{n}\left(x,\omega \right)$ are also obtained by a linear combination of the derivatives/integrals of the shifted functions Eup(x,ω) using the coefficients (A15) and (A16).

The exponential function $e^{2^{m}\omega x},\;m=0,1,\ldots ,n,\;n\in N$ on an interval $2^{-n}$ can be accurately represented by a linear combination $\left(n+2\right)\cdot 2^{n}$ of the basis function ${\mathrm{EFup}}_{n}\left(x,\omega \right),$ offset from one another by $2^{-n}$ in the form

$$e^{2^{m}\omega x}=\overset{\infty }{\underset{k=-\infty }{\sum }}\frac{e^{2^{m}\cdot \omega \cdot k\cdot \Delta x_{n}}}{A_{n,x}^{\left(m\right)}}\cdot {\mathrm{EFup}}_{n}\left(x-k\cdot \Delta x_{n},2^{n}\cdot \omega \cdot \Delta x_{n}\right);\;m=0,1,\ldots ,n$$

where the coefficients $A_{n,x}^{\left(m\right)}$ are

$$A_{n,x}^{\left(m\right)}=\overset{\left(n+2\right)\cdot 2^{n-1}}{\underset{i=-\left(n+2\right)\cdot 2^{n-1}}{\sum }}e^{2^{m}\cdot \omega \cdot i\cdot \Delta x_{n}}$$
